# Evaluation of the effectiveness of a 7-week minimal guided and unguided cognitive behavioral therapy-based stress-management APP for students

**DOI:** 10.1186/s12889-025-23399-4

**Published:** 2025-07-02

**Authors:** Elisabeth Margarete Weiss, Mona Harder, Siegmund Staggl, Bernhard Holzner, Verena Dresen, Markus Canazei

**Affiliations:** 1 Department of Psychology, University of Innsbruck, Innrain 52 f, Innsbruck, 6020 Austria; 2 Department of Psychiatry II - Institution for mental and psychosomatic illnesses, Medical University of Innsbruck, Innsbruck, 6020 Austria

**Keywords:** Web-based stress management intervention, Guided self-help, Emotion regulation, Coping skills

## Abstract

Stress during academic training is a significant issue for students, which intensified during the COVID-19 pandemic. Internet-based stress management programs are an attractive, low-threshold and cost-effective option for students at universities. The present randomized controlled intervention study examined the efficacy of a newly developed seven-week minimal guided (with optional e-Coach support) and unguided stress management training (iSMT) App based on cognitive behavioral therapy techniques (CBT) for university students. Participants (guided iSMT version: *n* = 118; unguided iSMT version: *n* = 123) were compared to a previous study’s passive psychoeducation group (*n* = 123) and waiting control group (*n* = 130). Participants were tested at pre- and post-intervention with the Depression Anxiety Stress Scale-21 (DASS-21), the Response Styles Questionnaire (RSQ) and the Emotion Regulation Questionnaire (ERQ). Both iSMT interventions significantly reduced perceived stress compared to the psychoeducation group and the waiting-list control group (all *p*s < .001; all ηp^2^ > .056), but no significant differences were found between the two versions of the iSMT. All intervention groups further showed improved emotion regulation (higher reappraisal) and coping (lower symptom-related rumination) strategies, with a small effect. These findings support iSMT as a cost-effective, accessible intervention for student stress management, offering benefits beyond traditional psychoeducation.

**Supplementary Information:**

The online version contains supplementary material available at 10.1186/s12889-025-23399-4.

## Background

In recent years, and particularly during the Covid-19 pandemic, the number of students suffering from mental illness has risen steadily (for a review, see [1]). Late adolescence and early adulthood in particular are considered vulnerable periods for the initial manifestation of various mental illnesses, which in more than half of cases (62.5%) manifest before the age of twenty-five [2]. Stress is one important risk factor for many mental disorders, such as depression, anxiety disorders, addictions and sleep disorders [3]. Typical stressors for students are high workload pressure, (oral) exams, writing papers or giving presentations in front of an audience (e.g., [4, 5]; for a review, see also [6]), but also non-academic stressors such as financial problems, part-time jobs, poor time management, interpersonal problems, lack of social support and moving away from familiar surroundings [7–9], are prevalent in this phase of life. Even in surveys conducted before the Covid-19 pandemic, a quarter [10] to half [11] of students reported high levels of stress. Various meta-analyses have shown that students’ stress levels have further increased during the Covid-19 pandemic, with up to two-thirds of students reporting high levels of stress [12, 13].

Despite the high prevalence of stress and mental health issues among students, few students are accessing support services [14–16]. In addition to structural barriers such as funding, lack of time and accessibility, attitudinal barriers such as prejudice, lack of openness in dealing with mental disorders, fear of judgment and labeling, and lack of confidence in the effectiveness of support services and the need to solve the problem oneself are the main reasons for the reluctance to use professional support services [17–19].

Internet-based programs are an attractive, low-threshold and cost-effective option for students at universities, particularly for preventive measures, for which the willingness to try and invest a lot of time and money is usually low [20, 21]. Students’ high level of digital literacy makes it easier for them to access and use online interventions [11], which is also reflected in previous studies in the high level of acceptance and user satisfaction with corresponding programs (e.g. [16, 20, 21]).

Research interest in the field of internet-based interventions has increased significantly during the Covid-19 pandemic and the associated need for social distancing [22]. Meta-analyses have demonstrated the effectiveness of internet-based interventions in students for a range of mental conditions and problems, including depression, anxiety, eating disorders and stress, with small to medium effects across different conditions [22]. These internet-based interventions are often based on the principles of cognitive behavioral therapy (CBT), and combined with acceptance and mindfulness concepts, skills training and mind–body interventions (e.g. meditation, mindfulness, breathing exercises or relaxation techniques; [23]). In terms of duration, internet-based stress interventions can be divided into short (1–4 weeks), moderate (5–8 weeks) and long (8 + weeks) interventions [23], with a short to moderate duration of online psychological interventions being recommended, especially for preventive stress reduction programs [24, 25].

The effectiveness of online interventions seems to be strongly related to the degree of direct, professional guidance by specialists or semi-skilled staff, so-called e-Coach [25–27]. In general, a distinction can be made between guided, minimally guided and unguided interventions [21, 28, 29]. In unguided interventions, participants receive no direct personal support. However, unguided internet-based therapies often incorporate brief automated messages aimed at enhancing adherence and reinforce participants’ progress through treatment, while still lacking additional personal support.

In guided interventions, an e-Coach provides support either synchronously (real-time interaction e.g. via chat or video) or asynchronously (communication that does not require both parties to be online simultaneously). Participants can send messages at any time and receive responses after a delay (e.g., per e-mail or chat). Due to the substantial variation in both the format (e.g. written support via e-mails, video consultations, additional group sessions, etc.) and duration of therapeutic support across studies (ranging from 1 to 14 h), guided interventions can be further categorized based on the degree of human involvement into minimally guided interventions and intensively guided interventions.

In minimally guided interventions, an e-Coach offers support through individualized or semi-standardized text messages or emails, either upon request or at regularly intervals (e.g. weekly). In addition, participants may contact an e-Coach via a chat function or telephone for brief support as needed, such as clarifying comprehension questions, providing feedback on assignments and progress, or encouraging participants continued engagement with the intervention.

In studies with intensively guided interventions, therapeutic support is provided more frequently (e.g., two or three times per week), or with a faster response time (e.g., within 24 h), compared to the support offered in a minimally guided intervention. The qualification of the e-Coaches appears to be of minor importance for the improvement of symptom severity [28]. This means that the educational background of the e-Coaches is less important than thorough training. Nevertheless, the use of psychology students as e-Coaches has shown positive effects in several studies [30–32]. Other important aspects for effective coaching are a high level of motivation on the part of the e-Coaches and the time and care with which the e-coaching is carried out [28].

Reviews suggest that guided interventions are more effective than unguided interventions, both in improving psychopathological symptoms [28, 33] and in reducing stress [25, 34]. Heber et al. [25] postulated that guided interventions achieve comparable stress reduction to traditional face-to-face interventions, although the extent of contact and synchronicity in guided online interventions varied greatly in previous studies [28]. In addition, adherence and user participation are higher in guided online interventions [35], which in turn correlates positively with the therapeutic success of online interventions [36]. However, there are also studies that have found no difference in effectiveness between guided and unguided interventions [24, 37]. It is of great importance to investigate the correlation and influence of different forms of support in more detail [38] because intervention procedures that are associated with a low time and financial investment are needed to improve individual stress management and resilience in students.

In a previous study [39], we were able to show that a seven-week app-based passive psychoeducational stress management program for students led to a significant improvement in the adaptive emotion regulation strategy reappraisal and a significant reduction in the dysfunctional coping style symptom-focused rumination compared to a waiting-list control group. However, the passive psychoeducation program did not reduce the stress level or the depression and anxiety scores compared to a waiting-list control group in students who were in a low, non-clinically relevant range of these clinical scales at the beginning of the study. Compared to passive psychoeducational interventions, which only provide information but do not instruct specific interventions such as relaxation exercises, active stress management training has also been shown to reduce stress, depressed mood or other psychological distress in students [40–45].

The present study aimed to assess the stress-modulation effect of a newly developed seven-week minimally guided and unguided internet-based active stress management training program (iSMT). Additionally, it examined the program’s impact of the iSMT on emotion regulation and coping strategies. Data from students participating in the two versions of the iSMT were compared with findings from Weiss et al. [39], which investigated the stress-modulating effects of a passive psychoeducational stress management program alongside a waiting-list control group.

We hypothesized that both the guided and unguided iSMT interventions would lead to a statistically significant reduction in perceived stress levels compared to the psychoeducation group and the waiting-list control group, with the minimally guided iSMT demonstrating the greatest effectiveness. Furthermore, we expected that both the iSMT program and the passive psychoeducational stress management program would enhance students’ adaptive emotion regulation strategies while reducing maladaptive strategies such as rumination, distraction and suppression, relative to the waiting-list control group.

## Methods

### Sample

It should be noted that the data analyses reported here originate from two separately conducted studies with different focuses: (i) *Psychoeducation study*: Psychoeducation program vs. waiting-list control group [39] and (ii) 2) *i-SMT study*: stress management training with e-Coach vs. stress management training without e-Coach. The two studies were conducted in different periods: the *Psychoeducation study* took place from May to October 2022, while data for the *iSMT study* were collected from January to May 2023. Both studies were approved by the Ethics Review Board of the University of Innsbruck.

The studies were conducted with German-speaking students from different Austrian and German universities. Recruitment was primarily carried out via the University of Innsbruck’s e-mail distribution list, and social media platforms. Bachelor students of psychology received course credits for participation in the studies.

### Measurements

#### Depression-anxiety-stress scales (DASS-21)

The Depression Anxiety Stress Scales (DASS-21; German version) were used to record the participants’ stress levels [46]. The original version was developed by Lovibond and Lovibond [47]. It is a self-assessment procedure with 21 items, seven of which cover each of the anxiety, depression and stress scales [47]. A period of seven days is considered retrospectively for the DASS-21 scales. Respondents have the opportunity to rate the items on a scale from 0 to 3 (0 = “*Did not apply to me at all*”; 3 = “*Applied to me very much or most of the time*”). In the *iSMT study*, only the DASS-21 *Stress* subscale was used. An example item for the DASS-21 *Stress* subscale is: “*I found it difficult to relax*”.

The three subscales demonstrate strong psychometric properties, including high internal consistency and validity [48]. The DASS-21 exhibits satisfactory convergent and discriminant validity (and is at least equivalent to standard assessment measures used in German-speaking regions [46].

In the present study, the internal consistency of the DASS-21 stress scale was high (Cronbach’s α = 0.87).

#### Response Styles Questionnaire (RSQ-D)

The German version of the Response Styles Questionnaire (RSQ-D) was used to determine individual coping strategies. The RSQ-D is a self-report questionnaire that is used to evaluate reactions to negative emotional situations such as stress, anger or sadness. The short version of the RSQ-D used by Kühner et al. [49] is based on the original version by Nolen-Hoesksema [50] and measures the three coping styles *Self-related Rumination*, *Symptom-focused Rumination*, and *Distraction*. Example items for the respective scales are:

- “*When I feel sad or depressed, I think about how alone I feel.*” (Self-related Rumination), “*When I feel sad or depressed, I think about how hard it is to concentrate.*” (Symptom-focused Rumination) and “*When I feel sad or depressed, I do something that made me feel better in the past.*” (Distraction). The short form of the RSQ-D used here consists of 23 items, which are to be rated by the participants on a four-point Likert scale (1 = “*almost never*”; 4 = “*almost always*”). The evaluation is carried out by summing the respective subscales. Eight items are assigned to the *Symptom-focused Rumination* subscale, while seven items make up the *Self-related Rumination* subscale and a further seven items represent the *Distraction* subscale. Regarding psychometric properties, the RSQ-D demonstrates both convergent and predictive validity for depressive symptom severity in both clinical (depressed patients) and non-clinical populations, as well as in relation to conceptually related scales [49]. The internal consistency of the RSQ-scales in the current study was sufficient (Symptom-focused Rumination: Cronbach’s α = 0.81; Self-related Rumination: Cronbach’s α = 0.77; Distraction: Cronbach’s α = 0.72).

#### Emotion Regulation Questionnaire (ERQ)

The Emotion Regulation Questionnaire (ERQ) [51] (German version [52]) is a self-evaluation questionnaire and contains ten items designed to record a preference for the two emotion regulation strategies *Reappraisal* (six items) and *Suppression* (four items). The items are answered on a Likert scale, with 1 being “*strongly disagree*” and 7 being “*strongly agree*”. All items, assigned to each of the two regulation strategies are averaged for the evaluation. Example items for the individual scales are: “*When I’m faced with a stressful situation, I make myself think about it in a way that helps me stay calm*.” (Reappraisal) and “*When I am feeling positive emotions, I am careful not to express them.*” (Suppression).

The ERQ demonstrates strong external validity across diverse cultural and demographic groups. Expressive suppression scores were positively correlated with psychological distress and alexithymia, while cognitive reappraisal scores were generally linked to adaptive outcomes [53]. Convergent and discriminant validity of the German version of the questionnaire result from its correlative relationships to other methods [52]. The internal consistency of the ERQ-scales in the current study was sufficient (Reappraisal: Cronbach’s α = 0.84; Suppression: Cronbach’s α = 0.78).

### Procedure

#### Implementation of the *Psychoeducation study*

A detailed description of the psychoeducation study can be found in the paper published by Weiss et. al. [39]. A total of 294 participants took part in the *Psychoeducation study* [39]. After the screening, all participants were randomly assigned to a seven-week psychoeducation group (*n* = 147) or a waiting-list control group (*n* = 147) stratified by age, gender and DASS-21 stress score. The participants in the psychoeducation group were provided with text-based materials once a week on Monday using the Quenza app (https://quenza.com), with a reading time of approx. 10–15 min per module. The content focus of the individual modules was in the same order: Week 1: Stress, Week 2: Daily structure, Week 3: Emotion regulation and problem solving, Week 4: Sleep, Week 5: Enjoyable activities, Week 6: Physical activities, Week 7: Nutrition. At the end of the week (on Fridays), an evaluation of the information material was obtained, in which the comprehensibility of the texts and the personal relevance of the topic that the participants perceived when working through the psychoeducation material were queried. Following the seven weeks of psychoeducational stress training, the participants received a link via email containing the questionnaires for the post-measurement of the study-relevant characteristics. In addition, the participants were asked to evaluate the training program. The waiting-list control group did not receive any psychoeducational information material during the 7 weeks. However, access to the psychoeducational material was activated for the participants in the waiting-list control group following the survey at the end of the 7 weeks. Finally, 253 students (*n* = 123 psychoeducation group; *n* = 130 waiting-list control group) participated fully in all relevant surveys and were included in the analyses. Exact details of this study can be found in Weiss et al. [39].

#### Implementation of the *iSMT study*

Participants received information about the background of the study and the stress management training program by email and on the start page of the care4stress app. The iSMT program was implemented with the CHES software [54] and all questionnaires were accessible digitally via the care4stress app or the associated website.

In previous studies, the extent of therapeutic support provided by an e-Coach varied widely and it has been suggested that this can influence the effectiveness of the internet-based interventions and the commitment of the participants [24, 26, 28, 33]. Therefore, in the present study the iSMT was developed as minimally guided versus an unguided version. The minimally guided iSMT version comprised written motivational messages at the end of each week and standardized specific feedback depending on the reported difficulties in the implementation of the transfer exercises. In addition, in the minimally guided intervention, asynchronous chat-based contact with an e-Coach was available on demand. Participants who wished to engage in active communication, ask questions, or provide feedback could send messages at any time and receive responses after a delay via chat.

In the unguided iSMT version the participants received a fully automated standardized motivational message at the end of the week, indicating whether they had successfully completed a module or had achieved a lower level of completion, without any additional opportunity for communication with an e-Coach.

Throughout the study, the e-Coach consistently monitored participants’ engagement with the exercises by tracking the number of logins and the time spent on the platform. To assess adherence, we utilized the e-Coach’s information on the successful completion of both mandatory and optional transfer exercises, as well as the number of logins related to these exercises and calculated the frequency of completed transfer exercises.

**Fig. 1:**
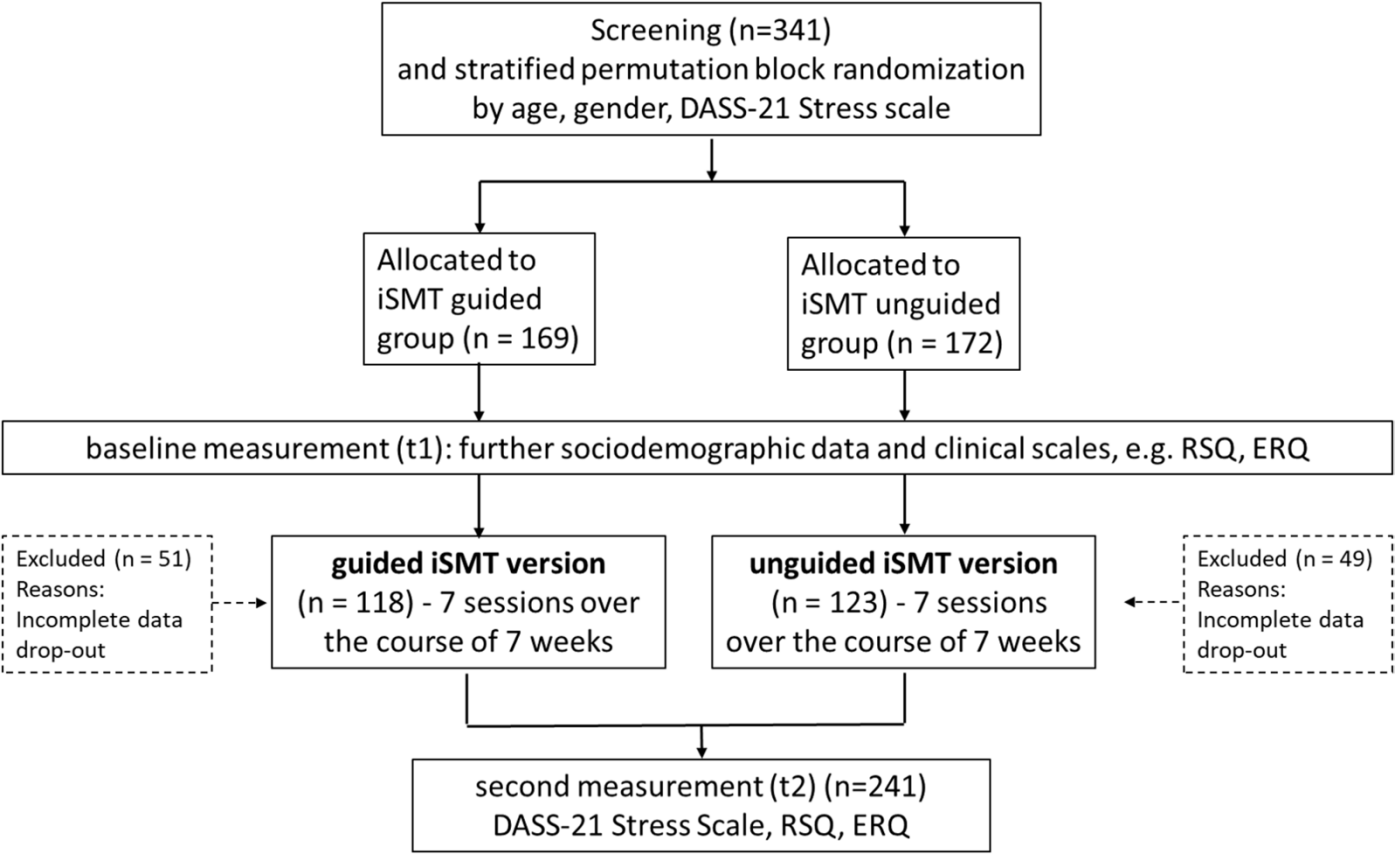
Flow diagram of the *iSMT study*

For an overview of study procedure of the *iSMT study*, including the questionnaires used, see Fig. 1.

After the screening, participants in the *iSMT study* were assigned to either the guided or unguided iSMT version using a stratified randomized procedure (graded to the following parameters and order: age, gender, stress level). In the *iSMT study*, 341 participants initially were included, of whom 169 were assigned to the guided iSMT version and 172 to the unguided iSMT version.

The participants then received a battery of questionnaires to collect sociodemographic data and clinical questionnaires. The subsequent seven-week stress management training program comprised six modules and a final outlook and summary module, which were completed consecutively. One week (Monday to Sunday) was scheduled for each of these modules. At the beginning of each week, the participants in the two *iSMT study* arms were provided with materials to explain the topic of the module. Briefly, text modules, case vignettes and short videos were used to familiarize participants with the various topics. Moreover, interactive elements (i.e. quizzes and reflection questions) were also part of the modules. On the same day, transfer exercises from the areas of cognitive behavioral therapy and mindfulness were activated in which the acquired knowledge could be applied in practice. These were to be carried out around three times over the course of the week in order to successfully complete the module. The mandatory exercises took about 10–15 min per day. If the mandatory exercise was not completed, the participants received a motivating message to start the training again next week. In addition, participants had the opportunity to complete further optional exercises and completing one optional exercise per week was recommended in order to deepen the training success. To increase adherence, participants were reminded via the app to complete questionnaires, quizzes and reflection questions and carry out planned transfer exercises. This notification function could be deactivated by the participants if required.

Furthermore, the program offered all students in the guided and unguided iSMT group access via a link to the psychoeducation program used in the study by Weiss et al. [39]. Additionally, at the beginning of each module a short introduction to the topic of each module was presented.

Table 1 describes the content of the individual modules with the respective transfer exercises. Each of these modules had a different thematic focus, but all had the common goal of providing the participants with specific stress management strategies.

**Table 1:** Thematic focus and transfer exercises of the seven modules of the iSMT program

Modules	Module description	Transfer exercises
Module 1	Stress and regeneration	stress diary; optional: breathing exercise
Module 2	Recovery and enjoyment	gratitude diary; optional: body scan, pleasure activities
Module 3	Structuring the day	ABC principle; optional: breathing exercise, 5–4-3–2-1 exercise
Module 4	Cognitive stress management strategies	Column technique; optional: nature recovery project, rumination diary
Module 5	Emotion regulation	Imagination of a safe place; optional: mindfulness exercise, emotion surfing
Module 6	Problem solving	Problem-solving training; optional: recovery project: time-out with colleagues
Module 7	Outlook and summary of the modules	no transfer exercises were provided

**Fig. 2:**
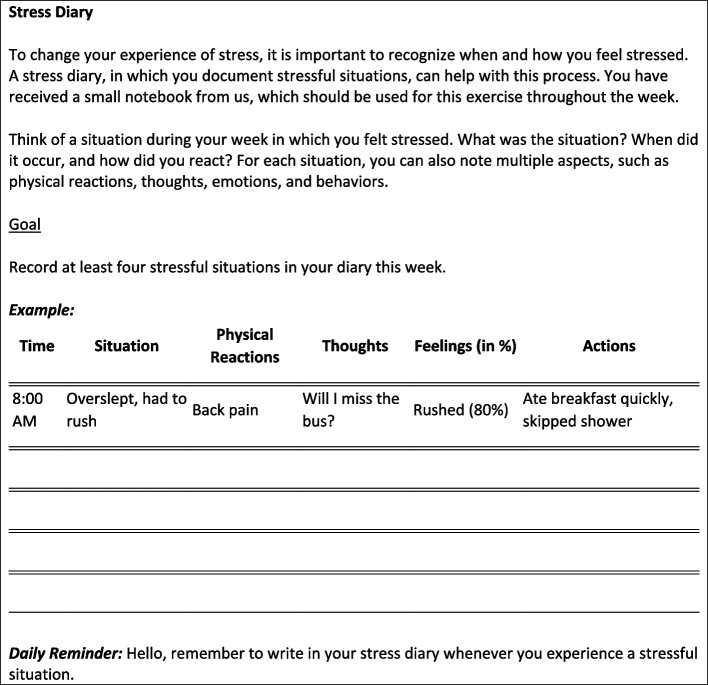
Example of a transfer exercise (Module 1)

Figure 2 gives an example of a transfer exercise from Module 1.

### Evaluation of each module

At the end of each week, the stress level was recorded using the DASS-21 Stress-subscale. In addition, each participant evaluated the adherence and satisfaction as well as problems with the individual exercises (e.g. I couldn’t concentrate on the exercise; it was too difficult, the exercise duration was too long etc.).

The unguided iSMT intervention included automated reminders and standardized weekly feedback regarding, whether the participants completed the transfer exercises successfully. If the participants did only one exercise or less they received a motivating message to start the training again next week. The unguided ISMT group had no opportunity to respond to this e-coach message.

Participants in the minimally guided iSMT group received automated reminders and a motivational message at the end of each week informing them whether a module had been completed successfully or less successfully, as well as an outlook on the content of the coming week. The messages contained matching emojis and were addressed to the respective person (salutation with surname). In addition, the e-Coach provided standardized specific feedback depending on the reported difficulties encountered in the transfer exercises e.g. “You have reported that you had problems with your posture during the breathing exercise? The breathing exercise can be performed in different postures (e.g. sitting, lying, standing). How did you manage to do the breathing exercise every day despite these problems?”. The participants were given access to a chat function to actively communicate with the e-Coach, ask questions or give feedback. Participants who wished to engage in active communication could send messages at any time and receive responses after a delay via chat.

From the 7th week onwards, the participants had the opportunity to complete the final questionnaire battery (second measurement t2) within 10 days.

Finally, 241 students (*n* = 118 guided iSMT version; *n* = 123 unguided iSMT version) participated in all surveys and were included in the per-protocol (PP) analyses.

### Statistics

Descriptive statistics are presented as means (M) and standard deviations (SD). To compare demographic variables (age, gender, the number of psychology students per group, and mental illness) between the four groups, either analysis of variance, chi-square test, or Fisher’s exact test were performed. Differences in the number of completed transfer exercises between the guided and unguided iSMT groups were determined by t-tests for two independent samples. Additionally, Pearson correlations analyses were conducted to examine the relationship between exercise frequency and changes in stress levels and coping strategies with change scores defined as the difference between post-treatment and baseline measurements.

Multivariate analysis of variance (MANOVAs) and univariate analysis of variance (ANOVAs) were performed to examine baseline group differences in the questionnaires, using the questionnaire scores as dependent variables. A two-way mixed (M)ANOVA was conducted to examine the questionnaire responses, using the between-subjects factor group (guided iSMT version, unguided iSMT version, psychoeducation group, waiting-list control group) and the within-subjects factor time (baseline measurement (t1), second measurement (t2)). The group x time interaction was of particular interest as it could reveal the effects of the different interventions on the parameters studied. Significant group x time interactions were examined by pairwise post hoc comparisons with Bonferroni correction. We tested for homogeneity of variances in the between-subjects factor using Box’s M test. Furthermore, we examined skewness and kurtosis to test for deviations from normal distribution in the dependent variables. Unless otherwise stated in the Sect. “Results”, we confirm that all these assumptions were met.

Data analysis included participants with complete baseline and second measurement data (PP analysis). To examine the proportion of subjects who dropped out from the study, a correlation analysis was performed to examine the relationship between the number of subjects in each study group and participation status (completers vs. drop-outs). Moreover, baseline scores on all subscales were compared separately for each study group between the completer sample and the drop-out sample by applying two independent samples t-tests with Bonferroni-corrections. Finally, an intent-to-treat (ITT) analysis was conducted using multiple imputation. The fully conditional specification method with predictive mean matching (10 nearest neighbours, 10 iterations) was used to generate 10 imputed datasets. Mixed-measures ANOVAs were performed on each imputed dataset, and Rubin’s rules were applied to pool test statistics.

All analyses were conducted with SPSS (version 29) and a significance level of 0.05 (two-tailed). We report partial eta-squared effect sizes (ηp^2^).

## Results

The final sample size for the PP analysis was *n* = 494 (guided iSMT version: *n* = 118; unguided iSMT version: *n* = 123; psychoeducation group: *n* = 123, waiting-list control group: *n* = 130).

A correlation analysis compared completion rates across the four intervention groups and showed a significant association (χ^2^(3,635) = 22.725; *p* < 0.001). The guided iSMT group had a significantly higher drop-out rate (30.2%) compared to the waiting-list control group (11.6%). No differences in completion rates were observed in the unguided iSMT and psychoeducation groups (see Table S1, Supplementary Materials).

The age range of the sample subjected to PP analysis (*n* = 494) was between 18 and 49 years (M ± SD: 22.24 ± 3.45). A total of 374 female, 117 male, and 3 gender-diverse students participated in this study. Of the participants, 90.6% (*n* = 444) were undergraduate students, while 8.8% (*n* = 43) were enrolled in a master’s program and 0.6% (*n* = 3) were students in a PhD program. Most students (87%) were studying psychology (*n* = 426). Regarding mental health, 183 participants (37%) reported that they were currently suffering from a mental illness or had a history of mental illness. Sociodemographic and study-related information for all groups is shown in Table 2.

**Table 2:** Sociodemographic and study-related information for all groups

		Guided iSMT version	Unguided iSMT version	Psycho-education group	Waiting-list control group
		(*n* = 118)	(*n* = 123)	(*n* = 123)	(*n* = 130)
Age; years	mean (SD)	22.2 (3.4)	22.3 (4.0)	22.0 (2.8)	22.4 (3.6)
	min.-max	18–44	18–49	18–34	18–36
Gender; n (%)	female	88 (74.6%)	92 (74.8%)	95 (77.2%)	99 (76.2%)
	male	29 (24.6%)	31 (25.2%)	28 (22.8%)	29 (22.3%)
	gender divers	1 (.8%)	0 (0%)	0 (0)	2 (1.5%)
University Course; n (%)	Psychology	108 (91.5%)	108 (87.8%)	95 (77.2%)	108 (83.1%)
	Other Courses	10 (8.5%)	15 (12.2%)	28 (22.8%)	22 (26.9%)
Level of Education; n (%)	Bachelor	104 (88%)	116 (94.3%)	103 (83.7%)	116 (89.2%)
	Master	14 (12%)	7 (5.7%)	18 (14.7%)	13 (10%)
	PhD	0 (0%)	0 (0%)	2 (1.6%)	1 (0.8%)
Past or current mental illness; n (%)	Yes	47 (39.8%)	38 (30.9%)	54 (43.9%)	44 (33.9%)
	No	71 (60.2%)	85 (69.1%)	69 (56.1%)	86 (66.1%)

*M* mean, *SD* standard deviation, *min.- max.* minimum – maximum

At baseline, there were no significant differences between the groups in the variables age (*F*(3, 490) = .262, *p* = .853, ηp^2^ = .002), gender (Fisher’s exact test: *p* = .852),education grade (Fisher’s exact test: *p* = .478) and past or current mental illness (χ^2^ (3) = 5.44, *p* = .142). However, the groups differed in the number of psychology students per group (χ^2^ (3) = 10.97, *p* = .012).

Moreover, the score in the DASS-21 *Stress* subscale did not differ between the four groups (*F*(3,490) = .433, *p* = .729, ηp^2^ = 0.03) at baseline. Based on two MANOVAs, there were no significant differences between the four groups in the three RSQ-D scales (*F*(9, 1470) = 1.681, *p* = .089, ηp^2^ = .010) and two ERQ scales (*F*(6,980) = .839, *p* = .549, ηp^2^ .005) at baseline. The means and standard deviations of all clinical scales at baseline and the second measurement are shown in Tables 4, 5 and 6.

A comparison of baseline scores between participants who completed the study and those who dropped out was performed across the four study groups (see Table S2, Supplementary Materials). Significant differences were observed in the DASS-21 *Stress* subscale only, where students who dropped out in both guided and unguided iSMT groups reported significantly higher baseline stress levels compared to students who completed the study (*p* = .042 and *p* = .006, respectively). No significant differences were found in the psychoeducation and waiting-list control groups for stress levels. For all other parameters (ERQ subscales and RSQ-D subscales), no statistically significant differences were observed between the completer and drop-out sample across any of the intervention groups (see Table S2, Supplementary Materials).

On average, participants in the guided iSMT group completed 20.52 (SD = 6.29) of the mandatory transfer exercises, whereas those in the unguided condition completed 20.23 (SD = 7.06). Regarding the optional modules, participants in the guided iSMT group completed 6.43 (SD = 5.63) of the optional transfer exercises, while those in the unguided condition completed 7.81 (SD = 7.96). No significant differences were observed between the two groups in the mean number of completed modules (mandatory modules: *t*(238) = .33, *p* = .370; optional modules: *t*(158) = −1.25, *p* = 0.107). Additionally, a significant positive correlation (*p* = .025) was found only between the frequency of optional transfer exercises and change-scores for the ERQ *Reappraisal* Subscale (see Table 3).

**Table 3:** Correlations between the frequency of mandatory and optional transfer exercises and the change-scores of the different scales

		**DASS-21** **Diff**	**RSQ_SELF** **Diff**	**RSQ_SYM** **Diff**	**RSQ_Diss** **Diff**	**ERQ_R** **Diff**	**ERQ_S** **Diff**
Mandatory Exercise	r p	-.046 .480	-.116 .074	.052 .420	-.004 .946	-.016 .806	.001 .992
Optional Exercise	r p	0.42 .597	-.053 .509	.044 .955	.107 .180	.177* .025	-0.33 .679

DASS-21 Diff = Change Score for the DASS-21 Stress subscale; RSQ_Self Diff: Change Score for the *Self-related Rumination Subscale*, RSQ_Sym Diff = Change Score for the *Symptom-focused Rumination* Subscale and RSQ_Diss Diff = Change Score for the *Distraction* Subscale of the Response Styles Questionnaire (RSQ-D); ERQ-R Diff Change Score for the *Reappraisal Subscale*, ERQ_S Diff = Change Score for the *Suppression* Subscales of the Emotion Regulation Questionnaire (ERQ)

### Stress evaluation

The means and standard deviations of the DASS-21 *Stress* subscale at the two measurement periods t1 and t2 are shown in Table 4.

**Table 4:** DASS-21 *Stress* subscale—means (M) and standard deviations (SD) at t1 and t2

**Groups**	*t1*	*t2*
	*M (SD)* *min.- max*	*M (SD)* *min.- max*
Guided iSMT version	7.51 (3.91) 0–21	5.57 (3.4) 0–16
Unguided iSMT version	7.86 (3.76) 0–20	5.89 (3.59) 0–16
Psychoeducation group	7.59 (4.18) 0–20	7.33 (4.25) 0–19
Waiting-list control group	7.31 (3.84) 0–18	7.1 (3.67) 0–19

*M* mean, *SD* standard deviation, *min.- max.* minimum – maximum

**Fig. 3:**
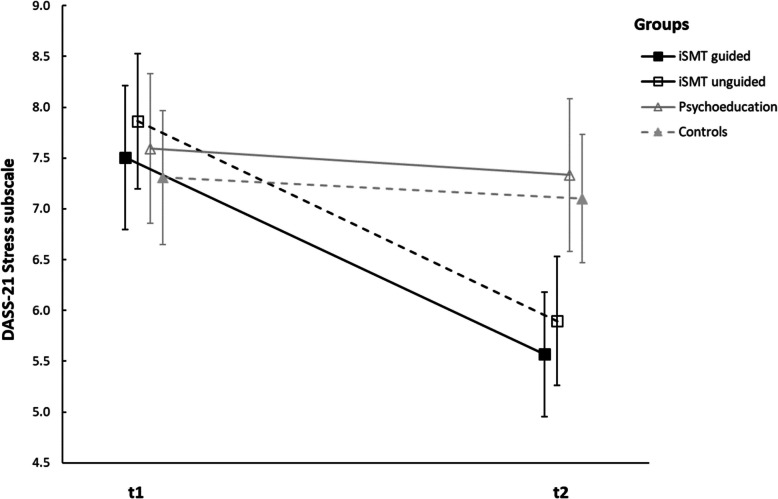
Changes of DASS-21 Stress subscale from t1 to t2. Note: the figure shows means and 95% confidence intervals of the means. A significant decrease in the DASS-21 *Stress* subscale was observed in the guided and unguided iSMT group

A mixed ANOVA for the DASS-21 *Stress* subscale showed a significant group x time interaction effect (*F*(3,490) = 8.059, *p* < .001, ηp^2^ = .047) and a significant main effect of time (*F*(1,490) = 39.036, *p* < .001, ηp^2^ = .074) but no main effect of group (*F*(3,490) = 1.786, *p* = .15, ηp^2^ = .011). Univariate variance analyses with repeated measures revealed a significant decrease in the DASS-21 stress score between the baseline and second measurement only in the guided iSMT Group (*F*(1,490) = 29.301, *p* < .001, ηp^2^ = .056) and the unguided iSMT group (*F*(1,490) = 31.543, *p* < 0.001, ηp^2^ = 0.06) but not for the psychoeducation group (*F*(1,490) = .511, *p* = .458, ηp^2^ = .001) and waiting-list control group (*F*(1,490) = .371, *p* = .543, ηp^2^ = .001). See Fig. 3 for an illustration of this effect.

### Coping style

The means and standard deviations of three subscales of the Response Styles Questionnaire (RSQ-D) at t1 and t2are shown in Table 5.

**Table 5:** Three subscales *Self-related Rumination*, *Symptom-focused Rumination* and *Distraction* of the Response Styles Questionnaire (RSQ-D) – mean (M) and standard deviation (SD) at t1 and t2

RSQ-D	Group	t1	t2
		M (SD) *min.- max*	M (SD) *min.- max*
*Self-related Rumination*	guided iSMT version	16.58 (4.22) 7–26	16.53 (3.92) 8–26
	unguided iSMT version	17.02 (3.84) 8–28	16.27 (3.75) 8–28
	Psychoeducation group	16.89 (4.37) 7–28	16.60 (4.31) 7–26
	Waiting-list control group	17.33 (4.19) 8–27	16.82 (4.33) 8–27
*Symptom-focused Rumination*	guided iSMT version	18.64 (4.71) 10–29	17.30 (4.05) 8–30
	unguided iSMT version	18.90 (4.75) 9–32	17.27 (4.44) 8–32
	Psychoeducation group	18.85 (5.06) 8–31	18.00 (5.10) 8–30
	Waiting-list control group	17.93 (4.58) 9–30	18.25 (5.09) 9–31
*Distraction*	guided iSMT version	18.18 (3.61) 10–29	19.71 (3.76) 11–29
	unguided iSMT version	17.91 (3.60) 8–26	19.08 (3.61) 10–32
	Psychoeducation group	17.75 (3.38) 8–25	18.15 (3.81) 9–27
	Waiting-list control group	17.25 (4.30) 8–28	17.71 (3.56) 10–30

*M* mean, *SD* standard deviation, *min.—max.* minimum – maximum, *RSQ-D* Response Styles Questionnaire

A multivariate mixed analysis of variance (MANOVA) for the three subscales of the RSQ-D (i.e., *Self-related Rumination*, *Symptom-focused Rumination*, and *Distraction*) revealed a significant interaction of group x time (*F*(9,1470) = 3.378, *p* < .001, ηp^2^ = .020) and a significant main effect of time (*F*(3,488) = 18.593, *p* < 0.001, ηp^2^ = 0.103). The main effect of group showed a trend towards significance (*F*(9,1470) = 1.859, *p* = .054, ηp^2^ = .011).

#### RSQ-D *Self-related Rumination* subscale

**Fig. 4:**
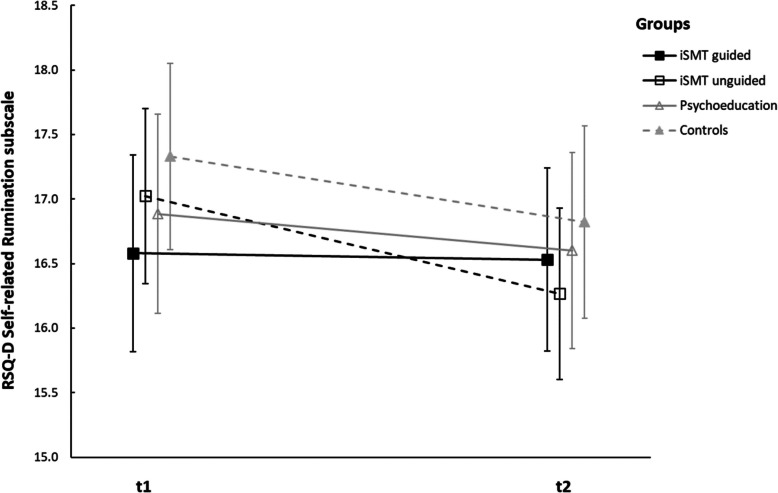
Changes of RSQ-D *Self-related Rumination* subscale from t1 to t2. Note: the figure shows means and 95% confidence intervals of the means

Univariate variance analyses with repeated measures revealed a significant main effect of time (*F*(1,490) = 8.248, *p* = .004, ηp^2^ = .017), but no significant main effect of group (*F*(3,490) = .446, *p* = .720, ηp^2^ = .003) and no significant interaction of group x time (*F*(3,490) = 1.162, *p* = .324, ηp^*2*^ = .007) in the *Self-related Rumination* subscale. See Fig. 4 for an illustration of this effect.

#### RSQ-D *Symptom-focused Rumination* subscale

**Fig. 5:**
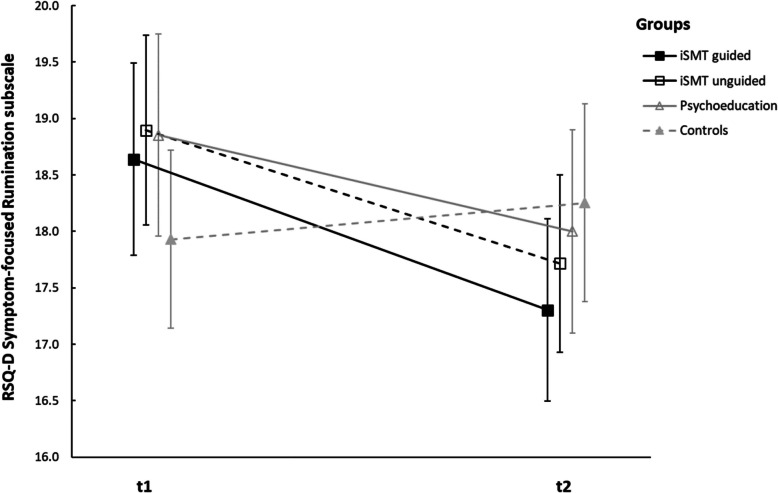
Changes of RSQ-D *Symptom-focused Rumination* subscale from t1 to t2. Note: the figure shows means and 95% confidence interval of the means. A significant decrease in the RSQ-D *Symptom-focused Rumination* Subscale was found in the guided and unguided iSMT group and the psychoeducation group

Univariate variance analyses with repeated measures revealed a significant interaction of group x time (*F*(3,490) = 5.304, *p* = .001, ηp^2^ = .031) and a main effect of time (*F*(1,490) = 21.263, *p* < 0.001, ηp^2^ = 0.042), but no significant main effect of group (*F*(3,490) = .263, *p* = .852, ηp^2^ = .002) in the *Symptom-focused Rumination* subscale. Post hoc tests showed a significant decrease in the *Symptom-focused Rumination* subscale from t1 to t2 for the guided iSMT group (*F*(1,490) = 15.616, *p* < .001, ηp^2^ = .031), unguided iSMT group (*F*(1,490) = 12.752, *p* < .001, ηp^2^ = .025) and the psychoeducation group (*F*(1,490) = 6.657, *p* = .01, ηp^2^ = .013), while the waiting-list control group showed no significant change (*F*(1,490) = 1.008, *p* = .316, ηp^2^ = .002), see Fig. 5 for an illustration of this effect.

#### RSQ-D *Distraction* subscale

**Fig. 6:**
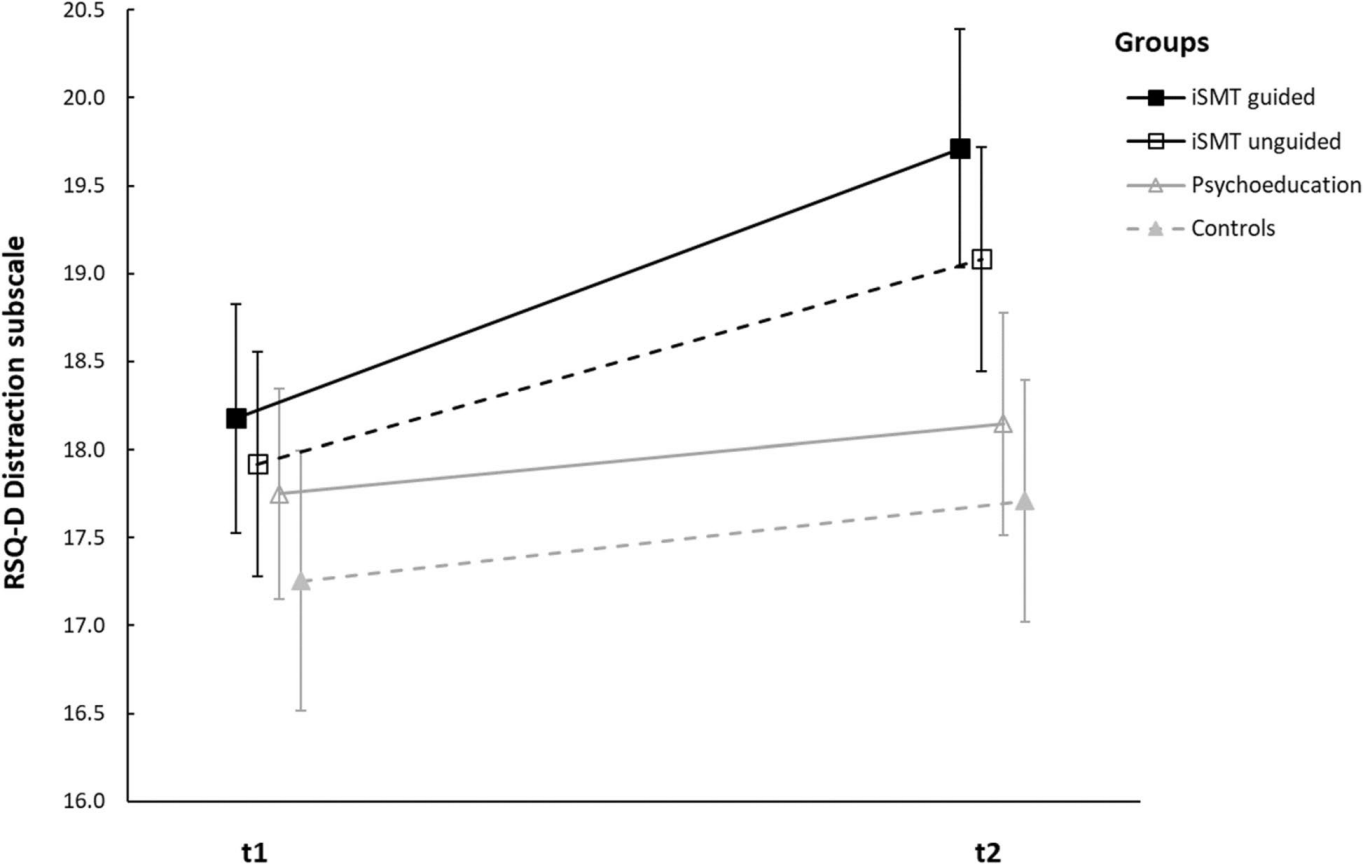
Changes of RSQ-D *Distraction* subscale from t1 to t2. Note: the figure shows means and 95% confidence intervals of the means. A significant increase in the RSQ-D *Distraction* subscale was found in the guided and unguided iSMT group

Univariate variance analyses with repeated measures revealed a significant interaction of group x time (*F*(3,490) = 3.675, *p* = .012, ηp^2^ = .022) and a main effect of time (*F*(1,490) = 38.040, *p* < .001, ηp^2^ = .072) and group *F*(3,490) = 4.407, *p* = .005, ηp^2^ = .026) in the *Distraction* subscale. Post hoc tests showed a significant increase in the *Distraction* subscale from baseline to the second measurement only for the guided iSMT group (*F*(1,490) = 27.185, *p* < .001, ηp^2^ = .053) and unguided iSMT group (*F*(1,490) = 16.317, *p* < .001, ηp^2^ = .032) but no significant increase for the psychoeducation group (*F*(1,490) = 1.908, *p* = .168, ηp^2^ = .004), and control group (*F*(1,490) = 2.617, *p* = .106, ηp^2^ = .005). Post hoc group comparisons showed significant higher distraction scores in the guided ISMT group compared to the waiting-list control group (*p* < .004). See Fig. 6 for an illustration of this effect.

### Emotion regulation

The means and standard deviations of the two subscales *Reappraisal* and *Suppression* of the Emotion Regulation Questionnaire (ERQ) at t1 and t2 are shown in Table 6.

**Table 6:** *Reappraisal* and *Suppression* subscales of the Emotion Regulation Questionnaire (ERQ) – mean (M) and standard deviation (SD) at t1 and t2

ERQ	Group	*t1*	*t2*
		*M (SD)* *min.- max*	*M (SD)* *min.- max*
*Reappraisal*	guided iSMT version	27.46 (5.79) 10–42	28.18 (5.76) 11–42
	unguided iSMT version Psychoeducation	26.91 (6.62) 6–41 26.03 (6.11) 8–39	28.25 (5.86) 6–41 28.39 (5.66) 10–42
	Waiting-list control group	26.08 (6.81) 6–41	26.61 (6.17) 8–41
*Suppression*	guided iSMT version	13.87 (4.93) 4–24	13.73 (4.58) 4–23
	unguided iSMT version	14.06 (5.11) 4–25	13.14 (4.96) 4–28
	Psychoeducation	13.59 (4.96) 4–25	13.25 (4.99) 4–26
	Waiting-list control group	13.65 (4.95) 4–26	13.92 (5.17) 4–27

*M* mean, *SD* standard deviation, *min.-max.* minimum–maximum

Finally, a multivariate mixed ANOVA was conducted for the ERQ subscales *Reappraisal* and *Suppression*. The analyses showed a significant group x time interaction (*F*(6,980) = 2.521, *p* = .02, ηp^2^ = .015) and a significant main effect of time (*F*(2,489) = 15.037, *p* < .001, ηp^2^ = .058) but no significant main effect of group (*F*(6,980) = 0.954, *p* = .456, ηp^2^ = .006).

#### ERQ *Reappraisal* subscale

**Fig. 7:**
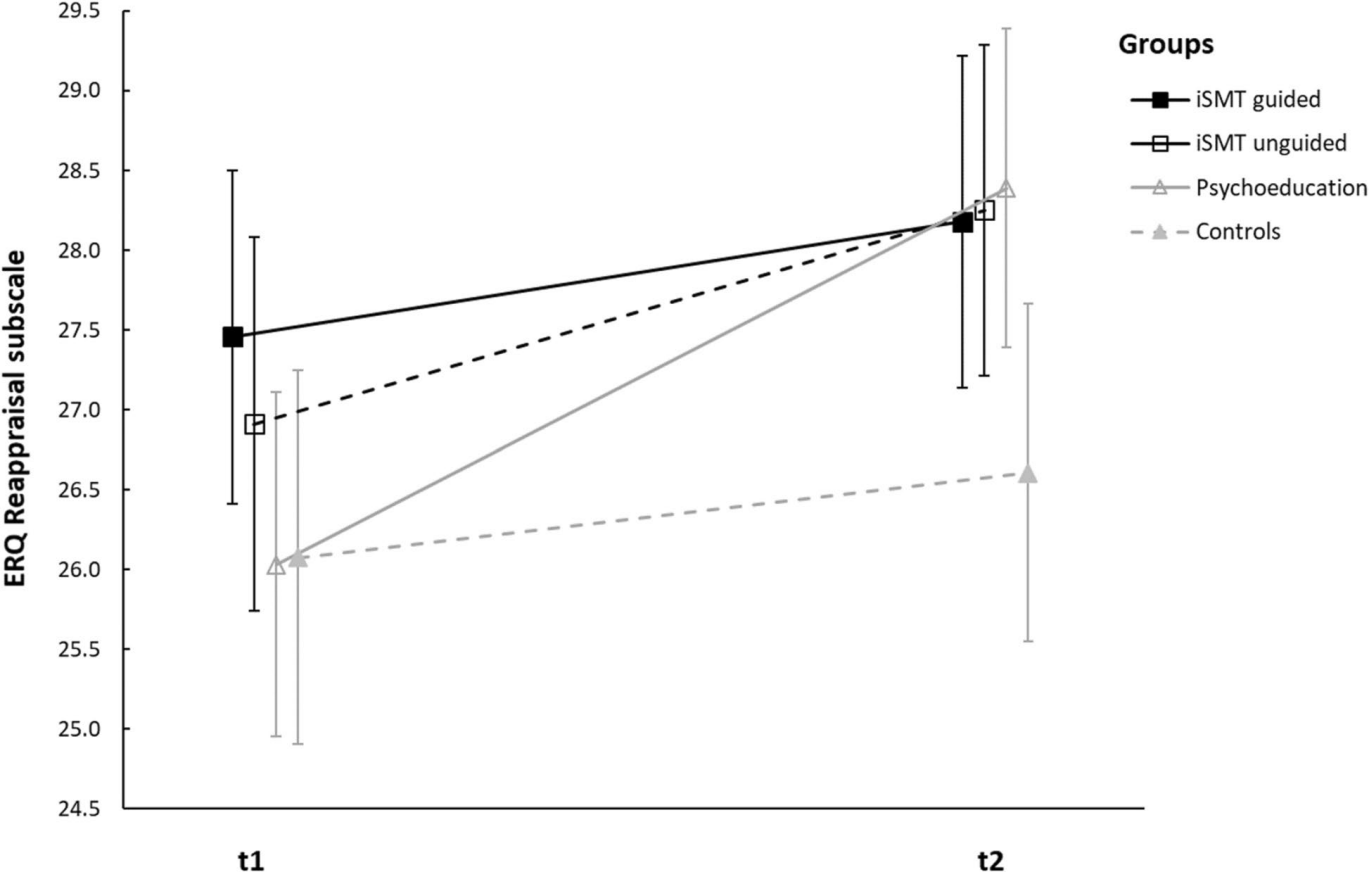
Changes of ERQ *Reappraisal* subscale from t1 to t2. Note: the figure shows means and 95% confidence intervals of the means. A significant increase in the ERQ *Reappraisal* subscale was found in the unguided iSMT and Psychoeducation group

An ANOVA with repeated measures reveals a significant group x time interaction in the ERQ *Reappraisal* subscale (*F*(3,490) = 3.057, *p* = .028, ηp^2^ = .018) and a significant main effect of time (*F*(1,490) = 27.531, *p* < .001, ηp^2^ = .053), but no significant main effect of group (*F*(3,490) = 1.727, *p* = .160, ηp^2^ = .010). A significant increase in *Reappraisal* was only found for the unguided iSMT group (*F*(1,490) = 8.037, *p* = .005, ηp^2^ = .016) and psychoeducation group (*F*(1,490) = 24.905, *p* = < .001, ηp^2^ = .048) but not for the guided iSMT group (*F*(1,490) = 2.246, *p* = .135, ηp^2^ = .005) and control group (*F*(1,490) = 1.334, *p* = .249, ηp^2^ = .003). See Fig. 7 for an illustration of this effect.

#### ERQ *Suppression* subscale

No significant effects were found for the ERQ *Suppression* subscale for the group x time interaction (*F*(3,490) = 2.012, *p* = .111, ηp^2^ = .012 and main effect of time (*F*(1,490) = 2.652, *p* = .104, ηp^2^ = .005) and group (*F*(3,490) = .191, *p* = .903, ηp^2^ = .001).

### ITT analyses

The ITT analysis revealed consistent interaction effects in the mixed ANOVAs compared to the PP analysis for the DASS-21 *Stress* subscale, the three subscales of the RSQ-D and the two subscales of the ERQ (see Table S3 and S4, Supplementary Materials).

## Discussion

The present study showed that the seven-week guided and unguided iSMTs resulted in a statistically significant reduction in perceived stress levels, with a small effect, compared to the psychoeducation group and the waiting-list control group, but no significant differences were found between the two versions of the iSMT. In addition, all intervention groups had a significant, albeit small, positive impact on various maladaptive or adaptive emotion regulation and coping strategies. Specifically, a significant reduction in symptom-focused rumination across all intervention groups compared to the control group could be shown. Additionally, a significant increase in the use of distraction as a coping strategy was observed exclusively in the two iSMT groups. Regarding emotion regulation, significant improvements in reappraisal were found in the unguided and psychoeducation groups, while no significant effects were observed on the Suppression subscale in any group.

The present study found no significant differences between the guided and unguided iSMT groups in terms of the number and frequency of completed mandatory and optional transfer exercises. This suggests that the presence of structured guidance via the e-Coach did not substantially impact participants’ engagement with the exercises. Both groups showed good engagement with the mandatory exercises but completed only few optional exercises. Interestingly, we found a significant positive correlation between the frequency of optional transfer exercises and improvements in cognitive reappraisal, as measured by the ERQ Scale Reappraisal scores.

### Perceived stress

Numerous studies indicated that internet-based interventions are not only effective in treating various mental disorders, but also can effectively reduce stress in university students, with small to moderate effects across conditions (e.g. meta-analysis [22]). Similarly, the present study demonstrated that both guided and unguided seven-week iSMT interventions significantly reduced perceived stress, with a small effect, compared to the psychoeducation and waiting-list control groups. However, no significant differences emerged between the two iSMT versions. Unlike psychoeducation, which primarily provides information and awareness about stress management, iSMT actively engages participants in mental skills training. Skill-based interventions, particularly those incorporating mindfulness and CBT techniques, enables individuals to develop cognitive and emotional regulation skills through repeated practice, potentially leading to more pronounced and lasting stress reduction effects, comparable to traditional face-to-face CBT interventions [25]. Such interventions provide accessible, scalable, and cost-effective solutions for individuals who may face barriers to in-person therapy, such as time constraints, stigma, or lack of resources. Importantly, they offer structured training that can be integrated into daily routines, facilitating long-term stress management.

Furthermore, reviews indicate that guided interventions generally yield greater effectiveness than unguided ones, both in alleviating psychopathological symptoms [28, 33] and in reducing stress [25, 34]. One potential reason for this difference is that guided interventions offer structured support, boosting motivation, adherence, and engagement, which enhances the application of stress management techniques in real life. In contrast, unguided interventions often have lower engagement and higher dropout rates, limiting their effectiveness.

The large number of publications already available on the subject of internet-based stress reduction shows a considerable degree of methodological heterogeneity. The existing literature exhibits fundamental differences, particularly with regard to the design of the intervention. For example, mindfulness-based methods [34, 55], cognitive-behavioral therapy methods [24, 56] or psychoeducational methods [57] were used. The studies also differed in terms of the measurement instrument used to record stress levels. These included the Perceived Stress Scale [16], the DASS-21 [56, 58] or the Perceived Stress Questionnaires [34, 59]. In addition, the duration of the studies varied considerably, ranging from two to twelve weeks. A meta-analysis showed that the intensity of the interventions studied, also varied greatly, ranging from two to forty sessions [22]. Despite considerable methodological heterogeneity in the existing literature (including the present *iSMT study*, most studies were able to demonstrate a low to moderate effect of stress management training on the subject’s stress experience compared to a control group [16, 22–25, 34, 57].

Another problem that affects the comparability of the current research is the different levels of stress experienced by the students. While some studies [34, 57], similar to the present study, included subjects with any level of stress, other studies focused on subjects with high levels of stress [16, 23, 58]. Reviews and meta-analyses point to the greater efficacy of indicated prevention, i.e., those targeting individuals with elevated baseline scores on clinical scales or pre-existing mental health conditions, compared to so-called universal prevention [23, 24, 26]. In the present study, the stress evaluations of the students at the beginning of the study were in a low, not clinically relevant range. While no significant reduction in stress levels was observed in the psychoeducation group [39], it was shown in the present study that the iSMT led to a significant reduction in perceived stress, even in students with low stress levels through the inclusion of interactive transfer exercises.

One issue that is heavily debated in the literature is the impact of the level of supervision and therapeutic support offered within the internet-based stress interventions. It has been suggested that this may influence the effectiveness of the internet-based stress interventions and the engagement of participants. Previous meta-analysis [25, 27, 28, 60] found a medium to high effect for guided programs, while unguided programs achieved only a small effect. The present study revealed no statistically significant difference between the minimally guided and the unguided iSMT group. The unguided version of the program included weekly standardized feedback on training success and reminders to complete questionnaires. It has been shown that the automatic reminders for inactivity already have a positive effect on adherence to internet-based programs [61]. In the present study, the e-Coach feedback in the minimally guided iSMT intervention was provided via chat messages with an asynchronous response pattern. Although the e-Coach is available for a chat, only 60.5% of participants in the guided group used this option at least once during the seven-week intervention. However, most of these chats related to organizational matters in connection with the training program or technical difficulties. It is unclear whether the on–off use of a chat function is sufficient to derive additional benefit from support provided by an e-Coach. Similarly, Küchler et al. (2023) [34] argue that the infrequent use of the e-Coach function (on average only twice in seven weeks) could be a factor in the relatively small discrepancy in the effectiveness of the two forms of support. In a study by Harrer et al. (2018) [62] with college students who had an elevated stress level, feedback on training success was only provided if the study participants considered it necessary. Only a small proportion of participants (5%) took advantage of the opportunity to receive individual feedback. This indicates that students have a low need for additional advice in app- or internet-based programs. The fact that most participants in the present study were psychology students likely explains why the unguided and minimally guided interventions produced similar results. Their prior knowledge, self-efficacy, and low reliance on external guidance may have minimized the usual advantages of guided interventions. In other populations, particularly clinical samples or individuals with less familiarity with stress management, the impact of guidance might be more pronounced. This assumption is supported by a meta-analysis by Koelen et al. [29], which showed a higher effectiveness of e-Coach supported internet-based interventions in clinical groups compared to technologically automated support.

### Coping and emotion regulation

In the present study, the effectiveness of a guided and an unguided iSMT was investigated in comparison to a passive psychoeducation and a waiting-list control group with regard to three different coping strategies. Previous studies found that symptom-focused and self-focused rumination would lead to increased stress levels, while distraction would have positive short-term effects on stress symptoms, but would only be partially effective in the long term [49]. In the present study, all intervention groups positively influenced emotion regulation and coping strategies, with a small effect. The iSMT training in the present study may have focused more on behavioral strategies, such as distraction, rather than on cognitive strategies, probably leading to a smaller effect in coping strategies. Previous studies using internet-based interventions directly targeting coping strategies and resilience found small to moderate effects in different coping strategies (e.g. [63]. A significant reduction in symptom-focused rumination was found in all intervention groups compared to the control group whereas a significant increase in the use of the coping strategy distraction was observed exclusively in the two iSMT groups. The iSMT likely included techniques that explicitly encouraged distraction as a coping mechanism, possibly through behavioral recommendations in the iSMT (e.g. recovery activities such as taking breaks with colleagues). Moreover, the structured nature of the iSMT may have reinforced the active use of distraction strategies, whereas other groups were not explicitly trained in this approach.

With regard to emotion regulation, only the unguided group and the psychoeducation group showed a significant improvement in reappraisal. Notably, the high proportion of psychology students in the sample (87%) may have influenced this finding, as they were likely already familiar with cognitive reappraisal and other coping strategies before the intervention. The unguided group and the psychoeducation group may have had more autonomy in applying reappraisal techniques. In contrast, within the guided interventions, the e-Coach provided standardized feedback based on participants reported difficulties with transfer exercises in each module. Therefore, the guided iSMT group may have been less inclined to experiment with different cognitive strategies, including reappraisal, due to the structured nature of the e-Coach feedback, which emphasized adherence to the iSMT program.

We found no effect on the *Suppression* subscale in any group. Emotion suppression is often considered a maladaptive regulation strategy, and none of the interventions seemed to encourage or discourage it explicitly. It’s possible that suppression function more as a trait-like characteristic and is less susceptible to short-term intervention effects compared to strategies such as distraction or reappraisal.

The results of the present study are consistent with those of previous research findings, which also only found improvements in individual coping strategies in some cases [29, 44, 55, 64, 65]. However, it should be noted that comparisons with other studies are limited due to the use of different measurement instruments that assess other coping strategies such as acceptance, denial, substance misuse and other emotion regulation strategies, including recognizing, understanding and accepting emotions, impulse control and access to emotion regulation strategies. Therefore, several studies using internet-based stress interventions and psychoeducation training have shown a very specific effect on different maladaptive or adaptive coping styles [41, 42, 57, 66]. In addition, these studies were not only designed very differently in terms of content, but some also had a specific focus on training adaptive coping strategies. In summary, the results of both this study show that both internet-based stress management training and psychoeducation training can be effective instruments of prevention by improving and strengthening individual coping strategies and thus promoting well-being and mental health.

The unequal drop-out rates across intervention groups highlight important patterns in participant retention. The guided iSMT group showed a significantly higher drop-out rate (30.2%) compared to the waiting-list control group (11.6%), which aligns with previous research indicating that guided web-based interventions often experience higher attrition rates than control conditions [67, 68]. This pattern may reflect the increased demands placed on participants in active intervention conditions. Notably, baseline stress levels were significantly higher among who dropped out of the study in both guided and unguided iSMT groups, suggesting that individuals with elevated stress may find it more challenging to adhere to internet-based interventions. This finding is consistent with a recently published review from Svärdman and colleagues (2022) [69], showing a significant association between increased stress severity and lower adherence rates. Taken together, this result underscores the challenge of maintaining consistent use of the digital interventions among highly stressed populations. Despite these differential drop-out patterns, the ITT analysis revealed consistent interaction effects across all outcome measures, indicating that both guided and unguided iSMT interventions generate similar changes in scores over time. This result strengthens the robustness of the reported intervention effects.

### Limitations

The present study is characterized by several limitations. It is important to note that the data analyses presented in this study come from two separate studies. An earlier study examined the efficacy of a passive psychoeducation program compared to a waiting-list control group [39]. In the subsequent *iSMT study*, which included both guided and unguided study arms, the students had access to the psychoeducation program that had already been evaluated in the previous study [39]. In addition, they received transfer exercises from the areas of cognitive behavioral therapy and mindfulness. Despite the implementation of stratified randomized allocation in both studies and the observation that the four groups showed no significant differences at baseline in terms of demographic and clinical variables, it is important to note that the studies were conducted at different time points. In particular, studies that use a pre-post design are prone to bias, especially in academic settings, as students may be exposed to different levels of stress, at the beginning of their studies or during the examination periods.

Second, it is also important to acknowledge that online interventions are susceptible to additional potential confounding factors that could influence the program use and questionnaire completion. In addition to the potential for disruptive environmental stimuli, fatigue or technical problems, these also include the possibility that some of the material is not read or worked through sufficiently or that the transfer exercises are not carried out correctly or carefully. A further limitation is that the two studies were conducted during different time periods, with the psychoeducation study taking place from May to October 2022 and data collection for the iSMT study occurring from January to May 2023. Given that COVID-19-related restrictions were gradually easing during this time, it is possible that differences in external stressors and daily life circumstances influenced participants’ stress levels and engagement with the interventions.

Third, more than two-thirds of the participants in the present sample are female and almost 90% are psychology students, which limits the generalizability of our results.

Psychology students likely had prior knowledge about stress, coping mechanisms, and therapeutic techniques, making them more capable of independently applying the intervention content. Their familiarity with stress management strategies might have reduced their need for additional guidance, explaining why there was no significant difference between the minimally guided and unguided groups.

Prior research indicates that reminders improve adherence [62], and since even the unguided group received standardized feedback and reminders, this may have been sufficient for psychology students to stay engaged. Because they already valued stress management, they may have been more intrinsically motivated to complete the intervention without needing external encouragement. Psychology students may have already been using cognitive reappraisal and other coping strategies before the intervention, leading to a potential ceiling effect with less room for improvement compared to participants from other backgrounds. This could explain why both groups benefited similarly, as they may have already had the ability to engage with and implement the training content effectively on their own. In a general population or clinical sample, individuals may not have the same level of psychological knowledge or motivation to apply stress-management techniques on their own. Therefore, the Generalizability to other population is limited.

Fourthly, a follow-up after three months was only carried out in the guided/unguided iSMT study, but not in the psychoeducation study. However, only few students took part in the follow-up evaluation (guided iSMT: *n* = 5; unguided iSMT: *n* = 12), so that no conclusions can be drawn about the sustainability of the observed effects. For example, certain procedures, such as relaxation techniques, become increasingly effective with practice. In addition, Bachelor students of psychology received course credits for participation in the study. This form of remuneration may have positively impacted participant adherence, since the prospect of earning these credits increased motivation to engage with the program, potentially leading to higher completion rates. However, while incentives may enhance adherence, they do not necessarily translate to greater intrinsic motivation or long-term behavioral change. Furthermore, as already discussed, a direct comparison with other studies is difficult due to the considerable methodological heterogeneity between the studies. Differences between studies include the measurement tools used, the content implementation, the duration of the intervention programs, and the form and amount of therapeutic support offered. It is also important to note that the ERQ and RSQ only encompass a subset of the possible emotion regulation or coping strategies. The selection of a suitable coping strategy often depends on the context [70]. The ERQ and RSQ do not adequately capture how individuals use these strategies in different contexts, how flexible they are in doing so, or how high their ability is to adapt their own strategies accordingly [71].

## Conclusions

The present study has shown that a seven-week guided and unguided online stress management training program had a positive impact on students’ stress experience compared to a passive psychoeducation program and a control group. In addition, all intervention groups showed a beneficial significant influence on various maladaptive or adaptive emotion regulation and coping strategies. No significant differences were found between the minimally guided and unguided iSMT versions in terms of their effectiveness in reducing stress levels in students with low baseline stress levels. The necessity of detailed feedback and interaction with an e-Coach for non-clinical student samples may depend on their prior knowledge and self-efficacy in stress management. While psychology students in this study showed little need for additional support, further research is needed to determine whether similar results apply to students from other disciplines. A personalized approach to the iSMT program, containing flexible selection of exercises and an optional e-Coach support, could enhance the program’s effectiveness.

## Supplementary Information

Supplementary Material 1.

## Data Availability

The datasets generated and/or analyzed during the current study are available from the corresponding author upon reasonable request.

## Abbreviations

DASS-21: Depression-anxiety-stress scales
ERQ: Emotion Regulation Questionnaire
iSMT: Internet-based stress management training program
ηp^2^: Partial eta-squared effect size
RSQ-D: Response Styles Questionnaire
t1: Baseline measurement
t2: Second measurement

## References

[1] Zarowski B, Giokaris D, Green O. Effects of the COVID-19 pandemic on university students’ mental health: a literature review. Cureus. 2024;16(2):e54032. 10.7759/cureus.54032. 38348205 10.7759/cureus.54032 PMC10859553

[2] Solmi M, Radua J, Olivola M, Croce E, Soardo L, Salazar de Pablo G, et al. Age at onset of mental disorders worldwide: large-scale meta-analysis of 192 epidemiological studies. Mol Psychiatry. 2022;27(1):281–95. 10.1038/s41380-021-01161-7. 34079068 10.1038/s41380-021-01161-7 PMC8960395

[3] American Psychiatric Association. Diagnostic and statistical manual of mental disorders (5th ed.). 2013. 10.1176/appi.books.9780890425596.

[4] Maser B, Danilewitz M, Guérin E, Findla L, Frank E. Medical student psychological distress and mental illness relative to the general population: a Canadian cross-sectional survey. Acad Med. 2019;94(11):1781–91. 10.1097/ACM.0000000000002958. 31436626 10.1097/ACM.0000000000002958

[5] Pozos-Radillo BE, Preciado Serrano M de L, Acosta-Fernández M, Aguilera-Velasco M de los Á, Delgado-García DD. Academic stress as a predictor of chronic stress in university students. Psicol Educ. 2014;20(1):47–52. 10.1016/j.pse.2014.05.006.

[6] Mofatteh M. Risk factors associated with stress, anxiety, and depression among university undergraduate students. AIMS Public Health. 2020;8(1):36–65. 10.3934/publichealth.2021004. 33575406 10.3934/publichealth.2021004 PMC7870388

[7] Delfino JP, Barragán E, Botella C, Braun S, Bridler R, Camussi E, et al. Quantifying insufficient coping behavior under chronic stress: a cross-cultural study of 1,303 students from Italy, Spain and Argentina. Psychopathology. 2015;48(4):230–9. 25967599 10.1159/000381400

[8] Logan B, Burns S. Stressors among young Australian university students: a qualitative study. J Am Coll Health. 2023;71(6):1753–60. 10.1080/07448481.2021.1947303. 34243688 10.1080/07448481.2021.1947303

[9] Pitt A, Oprescu F, Tapia G, Gray M. An exploratory study of students’ weekly stress levels and sources of stress during the semester. Act Learn High Educ. 2018;19(1):61–75. 10.1177/1469787417731194.

[10] Onieva-Zafra MD, Fernández-Muñoz JJ, Fernández-Martínez E, García-Sánchez FJ, Abreu-Sánchez A, Parra-Fernández ML. Anxiety, perceived stress and coping strategies in nursing students: a cross-sectional, correlational, descriptive study. BMC Med Educ. 2020;20(1):370. 10.1186/s12909-020-02294-z. 33081751 10.1186/s12909-020-02294-z PMC7574568

[11] Amanvermez Y, Karyotaki E, Cuijpers P, Salemink E, Spinhoven P, Struijs S, et al. Feasibility and acceptability of a guided internet-based stress management intervention for university students with high levels of stress: protocol for an open trial. Internet Interv. 2021;24:100369. 10.1016/j.invent.2021.100369. 33614413 10.1016/j.invent.2021.100369 PMC7878182

[12] Ochnik D, Rogowska AM, Kuśnierz C, Jakubiak M, Schütz A, Held MJ, et al. Mental health prevalence and predictors among university students in nine countries during the COVID-19 pandemic: a cross-national study. Sci Rep. 2021;11(1):18644. 10.1038/s41598-021-97697-3. 34545120 10.1038/s41598-021-97697-3 PMC8452732

[13] Xu T, Wang H. High prevalence of anxiety, depression, and stress among remote learning students during the COVID-19 pandemic: evidence from a meta-analysis. Front Psychol. 2023;13:1103925. 10.3389/fpsyg.2022.1103925. 36704682 10.3389/fpsyg.2022.1103925 PMC9871576

[14] Batchelor R, Pitman E, Sharpington A, Stock M, Cage E. Student perspectives on mental health support and services in the UK. J Furth High Educ. 2020;44(4):483–97.

[15] Grøtan K, Sund ER, Bjerkeset O. Mental health, academic self-efficacy and study progress among college students – the SHoT study, Norway. Front Psychol. 2019. 10.3389/fpsyg.2019.00045. 30733694 10.3389/fpsyg.2019.00045 PMC6354661

[16] Harrer M, Apolinário-Hagen J, Fritsche L, Salewski C, Zarski AC, Lehr D, et al. Effect of an internet- and app-based stress intervention compared to online psychoeducation in university students with depressive symptoms: results of a randomized controlled trial. Internet Interv. 2021;24:100374. 10.1016/j.invent.2021.100374. 33718001 10.1016/j.invent.2021.100374 PMC7932886

[17] Ebert DD, Mortier P, Kaehlke F, Bruffaerts R, Baumeister H, Auerbach RP, et al. Barriers of mental health treatment utilization among first-year college students: first cross-national results from the WHO world mental health international college student initiative. Int J Methods Psychiatr Res. 2019;28(2):e1782. 10.1002/mpr.1782. 31069905 10.1002/mpr.1782 PMC6522323

[18] Pretorius C, Chambers D, Coyle D. Young people’s online help-seeking and mental health difficulties: systematic narrative review. J Med Internet Res. 2019;21(11):e13873. 10.2196/13873. 31742562 10.2196/13873 PMC6891826

[19] Schnyder N, Panczak R, Groth N, Schultze-Lutter F. Association between mental health-related stigma and active help-seeking: systematic review and meta-analysis. Br J Psychiatry. 2017;210(4):261–8. 10.1192/bjp.bp.116.189464. 28153928 10.1192/bjp.bp.116.189464

[20] Amanvermez Y, Karyotaki E, Cuijpers P, Ciharova M, Donker M, Hurks P, et al. A guided, internet-based stress management intervention for university students with high levels of stress: feasibility and acceptability study. JMIR Form Res. 2023;7:e45725. 10.2196/45725. 37948106 10.2196/45725 PMC10674149

[21] Ebert DD, Cuijpers P, Muñoz RF, Baumeister H. Prevention of mental health disorders using internet- and mobile-based interventions: a narrative review and recommendations for future research. Front Psychiatry. 2017;8:116. 10.3389/fpsyt.2017.00116. 28848454 10.3389/fpsyt.2017.00116 PMC5554359

[22] Malinauskas R, Malinauskiene V. Meta-analysis of psychological interventions for reducing stress, anxiety, and depression among university students during the COVID-19 pandemic. Int J Environ Res Public Health. 2022;19(15):9199. 10.3390/ijerph19159199. 35954553 10.3390/ijerph19159199 PMC9368492

[23] Amanvermez Y, Zhao R, Cuijpers P, de Wit LM, Ebert DD, Kessler RC, et al. Effects of self-guided stress management interventions in college students: a systematic review and meta-analysis. Internet Interv. 2022;28:100503. 10.1016/j.invent.2022.100503. 35242591 10.1016/j.invent.2022.100503 PMC8861419

[24] Harrer M, Adam SH, Baumeister H, Cuijpers P, Karyotaki E, Auerbach RP, et al. Internet interventions for mental health in university students: a systematic review and meta-analysis. Int J Methods Psychiatr Res. 2019;28(2):e1759. 10.1002/mpr.1759. 30585363 10.1002/mpr.1759 PMC6877279

[25] Heber E, Ebert DD, Lehr D, Cuijpers P, Berking M, Nobis S, et al. The benefit of web- and computer-based interventions for stress: a systematic review and meta-analysis. J Med Internet Res. 2017;19(2):e32. 10.2196/jmir.5774. 28213341 10.2196/jmir.5774 PMC5336602

[26] Conley CS, Durlak JA, Shapiro JB, Kirsch AC, Zahniser E. A meta-analysis of the impact of universal and indicated preventive technology-delivered interventions for higher education students. Prev Sci. 2016;17(6):659–78. 10.1007/s11121-016-0662-3. 27225631 10.1007/s11121-016-0662-3

[27] Spek V, Cuijpers P, Nyklícek I, Riper H, Keyzer J, Pop V. Internet-based cognitive behaviour therapy for symptoms of depression and anxiety: a meta-analysis. Psychol Med. 2007;37(3):319–28. 10.1017/S0033291706008944. 17112400 10.1017/S0033291706008944

[28] Baumeister H, Reichler L, Munzinger M, Lin J. The impact of guidance on Internet-based mental health interventions — a systematic review. Internet Interv. 2014;1(4):205–15. 10.1016/j.invent.2014.08.003.

[29] Koelen JA, Vonk A, Klein A, de Koning L, Vonk P, de Vet S, et al. Man vs. machine: A meta-analysis on the added value of human support in text-based internet treatments (“e-therapy”) for mental disorders. Clin Psychol Rev. 2022;96:102179. 35763975 10.1016/j.cpr.2022.102179

[30] Kohtala A, Lappalainen R, Savonen L, Timo E, Tolvanen A. A four-session acceptance and commitment therapy-based intervention for depressive symptoms delivered by master’s degree level psychology students: a preliminary study. Behav Cogn Psychother. 2015;43(3):360–73. 10.1017/s135246581300096. 24229795 10.1017/S1352465813000969

[31] Lappalainen P, Granlund A, Siltanen S, Ahonen S, Vitikainen M, Tolvanen A, Lappalainen R. ACT internet-based vs face-to-face? A randomized controlled trial of two ways to deliver acceptance and commitment therapy for depressive symptoms: an 18-month follow-up. Behav Res Ther. 2014;61:43–54. 10.1016/j.brat.2014.07.006. 25127179 10.1016/j.brat.2014.07.006

[32] Räsänen P, Lappalainen P, Muotka J, Tolvanen A, Lappalainen R. An online guided ACT intervention for enhancing the psychological wellbeing of university students: a randomized controlled clinical trial. Behav Res Ther. 2016;78:30–42. 10.1016/j.brat.2016.01.001. 26848517 10.1016/j.brat.2016.01.001

[33] Karyotaki E, Efthimiou O, Miguel C, Bermpohl FMG, Furukawa TA, Cuijpers P, et al. Internet-based cognitive behavioral therapy for depression: a systematic review and individual patient data network meta-analysis. JAMA Psychiat. 2021;78(4):361–71. 10.1001/jamapsychiatry.2020.4364 PMC8027916 33471111

[34] Küchler AM, Schultchen D, Dretzler T, Moshagen M, Ebert DD, Baumeister H. A three-armed randomized controlled trial to evaluate the effectiveness, acceptance, and negative effects of StudiCare mindfulness, an internet- and mobile-based intervention for college students with no and “on demand“ guidance. Int J Environ Res Public Health. 2023;20(4):3208. 10.3390/ijerph20043208. 36833903 10.3390/ijerph20043208 PMC9965996

[35] Lehtimaki S, Martic J, Wahl B, Foster KT, Schwalbe N. Evidence on digital mental health interventions for adolescents and young people: systematic overview. JMIR Ment Health. 2021;8(4):e25847. 10.2196/25847. 33913817 10.2196/25847 PMC8120421

[36] Lattie EG, Adkins EC, Winquist N, Stiles-Shields C, Wafford QE, Graham AK. Digital mental health interventions for depression, anxiety, and enhancement of psychological well-being among college students: systematic review. J Med Internet Res. 2019;21(7):e12869. 10.2196/12869. 31333198 10.2196/12869 PMC6681642

[37] Nixon P, Boß L, Heber E, Ebert DD, Lehr D. A three-armed randomised controlled trial investigating the comparative impact of guidance on the efficacy of a web-based stress management intervention and health impairing and promoting mechanisms of prevention. BMC Public Health. 2021;21(1):1511. 10.1186/s12889-021-11504-2. 34353294 10.1186/s12889-021-11504-2 PMC8339390

[38] Bolinski F, Kleiboer A, Karyotaki E, Bosmans JE, Zarski A, Weisel KK, Ebert DD, Jacobi C, Cuijpers P, Riper H. Effectiveness of a transdiagnostic individually tailored internet-based and mobile-supported intervention for the indicated Prevention of Depression and Anxiety (ICARE Prevent) in Dutch college students: study protocol for a randomised controlled trial. Trials. 2018;19(1):1–13. 10.1186/s13063-018-2477-y. 29458407 10.1186/s13063-018-2477-y PMC5819200

[39] Weiss EM, Staggl S, Holzner B, Rumpold G, Dresen V, Canazei M. Preventive effect of a 7-week app-based passive psychoeducational stress management program on students. Behav Sci. 2024;14(3):180. 10.3390/bs14030180. 38540483 10.3390/bs14030180 PMC10968286

[40] Donker T, Griffiths KM, Cuijpers P, Christensen H. Psychoeducation for depression, anxiety and psychological distress: a meta-analysis. BMC Med. 2009;7:1–9. 20015347 10.1186/1741-7015-7-79 PMC2805686

[41] Hayes C, Morgan M. Evaluation of a psychoeducational program to help adolescents cope. J Youth Adolescence. 2005;34(2):111–21. 10.1007/s10964-005-3210-1.

[42] Jameson PR. The effects of a hardiness educational intervention on hardiness and perceived stress of junior baccalaureate nursing students. Nurse Educ Today. 2014;34(4):603–7. 10.1016/J.NEDT.2013.06.019. 23870691 10.1016/j.nedt.2013.06.019

[43] Jones LV. Enhancing psychosocial competence among Black women in college. Soc Work. 2004;49(1):75–84. 10.1093/SW/49.1.75. 14964520 10.1093/sw/49.1.75

[44] Steinhardt M, Dolbier C. Evaluation of a resilience intervention to enhance coping strategies and protective factors and decrease symptomatology. J Am Coll Health. 2008;56(4):445–53. 10.3200/JACH.56.44.445-45. 18316290 10.3200/JACH.56.44.445-454

[45] van Daele T, Welzijn S, EnGezin V, Hermans D, van Audenhove C, van den Bergh O. Stress reduction through psychoeducation: a meta-analytic review. Health Educ Behav. 2012;39(4):474–85. 10.1177/1090198111419202. 21986242 10.1177/1090198111419202

[46] Nilges P, Essau C. DASS. Depressions-Angst-Stress-Skalen - deutschsprachige Kurzfassung. Leibniz-Institut für Psychologie. 2021. 10.23668/psycharchives.4579.

[47] Lovibond SH, Lovibond PF. Depression Anxiety Stress Scales (DASS-21, DASS42). APA PsycTests. 1995. 10.1037/t01004-0.

[48] Henry JD, Crawford JR. The short-form version of the Depression Anxiety Stress Scales (DASS-21): construct validity and normative data in a large non-clinical sample. Br J Clin Psychol. 2005;44(Pt 2):227–39. 10.1348/014466505X29657. 16004657 10.1348/014466505X29657

[49] Kühner C, Huffziger S, Nolen-Hoeksema S. Response styles questionnaire - deutsche version; RSQ-D manual. Göttingen: Hogrefe; 2007.

[50] Nolen-Hoeksema S. Responses to depression and their effects on the duration of depressive episodes. J Abnorm Psychol. 1991;100(4):569–82. 10.1037/0021-843x.100.4.569. 1757671 10.1037//0021-843x.100.4.569

[51] Gross JJ, John OP. Individual differences in two emotion regulation processes: implications for affect, relationships, and well-being. J Pers Soc Psychol. 2003;85(2):348–62. 10.1037/0022-3514.85.2.348. 12916575 10.1037/0022-3514.85.2.348

[52] Abler B, Kessler H. Emotion regulation questionnaire—Eine deutschsprachige Fassung des ERQ von Gross und John. Diagnostica. 2009;55(3):144–52. 10.1026/0012-1924.55.3.144.

[53] Preece DA, Becerra R, Robinson K, Gross JJ. The emotion regulation questionnaire: psychometric properties in general community samples. J Pers Assess. 2020;102(3):348–56. 10.1080/00223891.2018.1564319. 30714818 10.1080/00223891.2018.1564319

[54] Holzner B, Giesinger JM, Pinggera J, Zugal S, Schöpf F, Oberguggenberger AS, et al. The Computer-based Health Evaluation Software (CHES): a software for electronic patient-reported outcome monitoring. BMC Med Inform Decis Mak. 2012;12:126. 23140270 10.1186/1472-6947-12-126 PMC3529695

[55] Sanilevici M, Reuveni O, Lev-Ari S, Golland Y, Levit-Binnun N. Mindfulness-based stress reduction increases mental wellbeing and emotion regulation during the first wave of the COVID-19 pandemic: a synchronous online intervention study. Front Psychol. 2021;12:720965. 10.3389/fpsyg.2021.720965. 34858260 10.3389/fpsyg.2021.720965 PMC8631924

[56] Yusufov M, Nicoloro-SantaBarbara J, Grey NE, Moyer A, Lobel M. Meta-analytic evaluation of stress reduction interventions for undergraduate and graduate students. Int J Stress Manage. 2019;26(2):132–45. 10.1037/str0000099.

[57] Bani Ahmad T, Meriç M. The effect of an online psychoeducational stress management program on international students’ ability to cope and adapt. Perspect Psychiatr Care. 2021;57(4):1673–84. 10.1111/ppc.12735. 33586178 10.1111/ppc.12735

[58] Rackoff GN, Fitzsimmons-Craft EE, Taylor CB, Eisenberg D, Wilfley DE, Newman MG. A randomized controlled trial of internet-based self-help for stress during the COVID-19 pandemic. J Adolesc Health. 2022;71(2):157–63. 10.1016/j.jadohealth.2022.01.227. 35351353 10.1016/j.jadohealth.2022.01.227 PMC8813578

[59] Fliege H, Rose M, Arck P, Levenstein S, Klapp BF. Validierung des “Perceived Stress Questionnaire“ (PSQ) an einer deutschen Stichprobe. Diagnostica. 2001;47(3):142–52. 10.1026/0012-1924.47.3.142.

[60] Andersson G, Cuijpers P. Internet-based and other computerized psychological treatments for adult depression: a meta-analysis. Cogn Behav Ther. 2009;38(4):196–205. 10.1080/16506070903318960. 20183695 10.1080/16506070903318960

[61] Zagorscak P, Heinrich M, Sommer D, Wagner B, Knaevelsrud C. Benefits of individualized feedback in internet-based interventions for depression: a randomized controlled trial. Psychother Psychosom. 2018;87(1):32–45. 10.1159/000481515. 29306945 10.1159/000481515

[62] Harrer M, Adam SH, Fleischmann RJ, Baumeister H, Auerbach R, Bruffaerts R, et al. Effectiveness of an internet- and app-based intervention for college students with elevated stress: randomized controlled trial. J Med Internet Res. 2018;20(4):e136. 10.2196/jmir.9293. 29685870 10.2196/jmir.9293 PMC5938594

[63] Herrero R, Franke M, Gorlich D, Garcia-Palacios A, Banos R, Jacobi C, Bergeri T, Schaub MB, Kriegeri T, Ebert DD, Botella C. Efficacy of the internet-based intervention “Cultivating our resilience” (CORE) for improving resilience and coping strategies in university students: a randomized controlled trial. Internet Interv. 2025;39:100811. 10.1016/j.invent.2025.100811. 40161472 10.1016/j.invent.2025.100811 PMC11954792

[64] Long R, Halvorson M, Lengua LJ. A mindfulness-based promotive coping program improves well-being in college undergraduates. Anxiety Stress Coping. 2021;34(6):690–703. 10.1080/10615806.2021.1895986. Lovibond SH, Lovibond PF. Depression Anxiety Stress Scales (DASS-21, DASS-42). APA PsycTests. 1995. 10.1037/t01004-000. 33719757 10.1080/10615806.2021.1895986

[65] Stächele T, Domes G, Wekenborg M, Penz M, Kirschbaum C, Heinrichs M. Effects of a 6-week internet-based stress management program on perceived stress, subjective coping skills, and sleep quality. Front Psychiatry. 2020;11:463. 10.3389/fpsyt.2020.00463. 32523554 10.3389/fpsyt.2020.00463 PMC7261857

[66] McCarthy B, Trace A, O’Donovan M, O’Regan P, Brady-Nevin C, O’Shea M, et al. Coping with stressful events: a pre-post-test of a psycho-educational intervention for undergraduate nursing and midwifery students. Nurse Educ Today. 2018;61:273–80. 10.1016/j.nedt.2017.11.034. 29288960 10.1016/j.nedt.2017.11.034

[67] Crutzen R, Viechtbauer W, Spigt M, Kotz D. Differential attrition in health behaviour change trials: a systematic review and meta-analysis. Psychol Health. 2014;30(1):122–34. 10.1080/08870446.2014.953526. 10.1080/08870446.2014.953526 25109224

[68] de Miquel C, Haro JM, van der Feltz-Cornelis CM, Ortiz-Tallo A, Chen T, Sinokki M, et al. Differential attrition and engagement in randomized controlled trials of occupational mental health interventions in person and online: a systematic review and meta-analysis. Scand J Work Environ Health. 2024;50(8):588–601. 10.5271/sjweh.4173. 39072699 10.5271/sjweh.4173 PMC11616721

[69] Svärdman F, Sjöwall D, Lindsäter E. Internet-delivered cognitive behavioral interventions to reduce elevated stress: a systematic review and meta-analysis. Internet Interv. 2022;29:100553. 10.1016/j.invent.2022.100553. 35781929 10.1016/j.invent.2022.100553 PMC9240371

[70] Fischer R, Scheunemann J, Moritz S. Coping strategies and subjective well-being: context matters. J Happiness Stud. 2021;22(8):3413–34. 10.1007/s10902-021-00372-7.

[71] Bettis AH, Burke TA, Nesi J, Liu RT. Digital technologies for emotion-regulation assessment and intervention: a conceptual review. Clin Psychol Sci. 2022;10(1):3–26. 10.1177/21677026211011982. 35174006 10.1177/21677026211011982 PMC8846444

