# Supplementary material for: Evaluation of the effectiveness of a 7-week minimal guided and unguided cognitive behavioral therapy-based stress-management APP for students
Source: BMC Public Health. 2025 Jul 2;25:2266. doi: 10.1186/s12889-025-23399-4 (PMC12219889; doi:10.1186/s12889-025-23399-4)
Supplement: Supplementary file 1 — Supplementary Material 1. [file 12889_2025_23399_MOESM1_ESM.docx]

**Evaluation of the effectiveness of a 7-week minimal guided and unguided cognitive behavioral therapy-based stress-management APP for students.**

**Supplementary Materials**

Table S1. **Correlation analysis**

|  | **completer sample (n=494)** | **drop-out sample (n=141)** |
| --- | --- | --- |
| guided iSMT, n(%), stdR | 118 (69.8%), -1.2 | 51 (30.2%), 2.2* |
| unguided iSMT, n(%), stdR | 123 (71.5%), -0.9 | 49 (28.5%), 1.7 |
| psychoeducation, n(%), stdR | 123 (83.7%), 0.8 | 24 (16.3%), -1.5 |
| WL control, n(%), stdR | 130 (88.4%), 1.5 | 17 (11.6%), -2.7* |

Note: n= sample size, % = proportion of subjects per study arm completing and dropping out; stdR = standardized residuals; stdR > 1.96 or stdR < -1.96 indicate significant cell frequency; WL control = waiting-list control group.

Table S2. **Comparison of baseline scores between the completer (n=494) and drop-out (n=141) samples.**

|  | **completer sample**, M(SD) | **drop-out sample**,  M(SD) | **Test-statistics**  (Bonferroni-corrected) |
| --- | --- | --- | --- |
| **DASS 21 - *STRESS* subscale** | | | |
| guided iSMT | 7.50 (3.91) | 8.88 (4.09) | *p* = .042* |
| unguided iSMT | 7.86 (3.76) | 9.76 (4.82) | *p* = .006* |
| psychoeducation | 7.59 (4.18) | 8.25 (3.95) | *p* = .466 |
| WL control | 7.31 (3.84) | 8.59 (4.53) | *p* = .219 |
| **ERQ - *Suppression* subscale** | | | |
| guided iSMT | 13.88 (4.93) | 14.04 (4.87) | *p* = .847 |
| unguided iSMT | 14.06 (5.11) | 14.22 (5.89) | *p* = .849 |
| psychoeducation | 13.59 (4.96) | 14.29 (5.01) | *p* = .531 |
| WL control | 13.65 (4.95) | 14.29 (4.96) | *p* = .623 |
| **ERQ - *Reappraisal* subscale** | | | |
| guided iSMT | 27.46 (5.80) | 25.84 (6.20) | *p* = .133 |
| unguided iSMT | 26.91 (6.62) | 25.45 (6.62) | *p* = .086 |
| psychoeducation | 26.03 (6.11) | 27.96 (7.75) | *p* = .178 |
| WL control | 26.08 (6.81) | 25.83 (5.38) | *p* = .677 |
| **RSQ-D - *Symptom-related Rumination* subscale** | | | |
| guided iSMT | 18.64 (4.71) | 19.76 (5.26) | *p* = .167 |
| unguided iSMT | 18.90 (4.75) | 19.94 (5.05) | *p* = .205 |
| psychoeducation | 18.85 (5.06) | 18.75 (5.48) | *p* = .924 |
| WL control | 17.93 (4.58) | 17.53 (4.65) | *p* = .749 |
| **RSQ-D - *Self-related Rumination* subscale** | | | |
| guided iSMT | 16.58 (4.22) | 16.67 (4.20) | *p* = .901 |
| unguided iSMT | 17.02 (3.84) | 17.04 (3.45) | *p* = .980 |
| psychoeducation | 16.89 (4.37) | 16.13 (5.03) | *p* = .410 |
| WL control | 17.33 (4.19) | 16.12 (3.55) | *p* = .329 |
| **RSQ-D - *Distraction* subscale** | | | |
| guided iSMT | 18.18 (3.61) | 17.24 (4.46) | *p* = .141 |
| unguided iSMT | 17.92 (3.60) | 16.71 (3.49) | *p* = .062 |
| psychoeducation | 17.75 (3.38) | 18.88 (4.49) | *p* = .185 |
| WL control | 17.25 (4.29) | 18.94 (3.40) | *p* = .086 |

Note: M = mean; SD = standard deviation; Test-statistics = test statistics of two independent sample t-tests, including Bonferroni-correction for multiple comparisons; DASS-21 = Depression-Anxiety-Stress Scale; ERQ = Emotion Regulation Questionnaire; RSQ-D = Responses Styles Questionnaire; WL control = waiting-list control group; * significant difference between samples.

Table S3. **Descriptive statistics comparing ratings at t1 and t2 between the completer and intent-to-treat (ITT) samples.**

| **groups** | **completer sample (n=494)** | | **ITT sample (n=635)** | |
| --- | --- | --- | --- | --- |
|  | **t1 – M(SD)** | **t2 – M(SD)** | **t1 – M*(SD*)** | **t2 – M*(SD*)** |
| **DASS-21 – *Stress subscale*** | | | | |
| guided iSMT | 7.51 (3.91) | 5.57 (3.4) | 7.92 (3.39) | 5.47 (3.76) |
| unguided iSMT | 7.86 (3.76) | 5.89 (3.59) | 8.40 (3.36) | 5.77 (3.74) |
| psychoeducation | 7.59 (4.18) | 7.33 (4.25) | 7.70 (3.63) | 7.27 (3.63) |
| WL control | 7.31 (3.84) | 7.1 (3.67) | 7.46 (3.79) | 7.05 (3.53) |
| **ERQ - *Suppression*** | | | | |
| guided iSMT | 13.87 (4.93) | 13.73 (4.58) | 13.92 (4.20) | 13.04 (4.49) |
| unguided iSMT | 14.06 (5.11) | 13.14 (4.96) | 14.11 (4.16) | 12.70 (4.30) |
| Psychoeducation | 13.59 (4.96) | 13.25 (4.99) | 13.70 (4.51) | 13.08 (4.56) |
| WL control | 13.65 (4.95) | 13.92 (5.17) | 13.73 (4.51) | 13.88 (4.57) |
| **ERQ – *Reappraisal*** | | | | |
| guided iSMT | 27.46 (5.79) | 28.18 (5.76) | 26.47 (5.35) | 28.21 (4.09) |
| unguided iSMT | 26.91 (6.62) | 28.25 (5.86) | 26.21 (5.31) | 27.93 (5.05) |
| psychoeducation | 26.03 (6.11) | 28.39 (5.66) | 26.35 (5.74) | 28.41 (5.19) |
| WL control | 26.08 (6.81) | 26.61 (6.17) | 26.16 (5.74) | 26.81 (5.20) |
| **RSQ-D - *Symptom-related Rumination*** | | | | |
| guided iSMT | 18.64 (4.71) | 17.30 (4.05) | 18.98 (4.05) | 17.66 (4.69) |
| unguided iSMT | 18.90 (4.75) | 17.27 (4.44) | 19.19 (4.02) | 17.98 (5.17) |
| psychoeducation | 18.85 (5.06) | 18.00 (5.10) | 18.84 (4.34) | 17.86 (4.87) |
| WL control | 17.93 (4.58) | 18.25 (5.09) | 17.88 (4.34) | 18.03 (4.85) |
| **RSQ-D - *Self-related Rumination*** | | | | |
| guided iSMT | 16.58 (4.22) | 16.53 (3.92) | 16.61 (3.44) | 16.37 (4.27) |
| unguided iSMT | 17.02 (3.84) | 16.27 (3.75) | 17.03 (3.42) | 16.47 (3.91) |
| psychoeducation | 16.89 (4.37) | 16.60 (4.31) | 16.76 (3.83) | 16.55 (3.83) |
| WL control | 17.33 (4.19) | 16.82 (4.33) | 17.08 (3.69) | 16.69 (3.78) |
| **RSQ-D - *Distraction*** | | | | |
| guided iSMT | 18.18 (3.61) | 19.71 (3.76) | 17.89 (3.19) | 19.53 (3,12) |
| unguided iSMT | 17.91 (3.60) | 19.08 (3.61) | 17.58 (3.16) | 18.95 (3.13) |
| psychoeducation | 17.75 (3.38) | 18.15 (3.81) | 17.93 (3.42) | 18.36 (3.32) |
| WL control | 17.25 (4.30) | 17.71 (3.56) | 17.75 (3.42) | 18.10 (3.31) |

Note: M = mean; SD = standard deviation; Statistics = two independent sample t-test; ITT sample = multiple imputation was applied to generate ten data sets; Rubin’s rules were applied to derive the pooled means (M*) and pooled standard deviations (SD*) across the ten data sets; DASS-21 = Depression-Anxiety-Stress Scale; ERQ = Emotion Regulation Questionnaire; RSQ-D = Responses Styles Questionnaire; WL control = waiting-list control group.

Table S4. **Pooled test statistics for the mixed ANOVAs of the ten imputed data sets.**

|  | **pooled F-statistic** | **pooled p-value** | **significant interaction effects** |
| --- | --- | --- | --- |
| **DASS-21 – *Stress subscale*** | | |  |
| Time | F(1,631) = 88.084 | *p* < .001 |  |
| Group | F(3,631) = 1.669 | *p* = .173 |  |
| Time x Group | F(3,631) = 15.429 | *p* < .001 | guided iSMT ↓, unguided iSMT ↓ |
| **ERQ – *Suppression*** | | |  |
| Time | F(1,631) = 19.885 | *p* = .030 |  |
| Group | F(3,631) = 0.266 | *p* = .850 |  |
| Time x Group | F(3,631) = 2.948 | *p* = .085 | n.a. |
| **ERQ – *Reappraisal*** | | |  |
| Time | F(1,631) = 47.195 | *p* < .001 |  |
| Group | F(3,631) = 1.172 | *p* = .321 |  |
| Time x Group | F(3,631) = 3.114 | *p* = .038 | unguided iSMT ↑, PE ↑ |
| **RSQ-D - *Symptom-related Rumination*** | | |  |
| Time | F(1,631) = 27.962 | *p* < .001 |  |
| Group | F(3,631) = 0.491 | *p* = .691 |  |
| Time x Group | F(3,631) = 4.399 | *p* = .011 | guided iSMT ↓, unguided iSMT ↓, PE ↓ |
| **RSQ-D - *Self-related Rumination*** | | |  |
| Time | F(1,631) = 7.055 | *p* = .049 |  |
| Group | F(3,631) = 0.320 | *p* = .810 |  |
| Time x Group | F(3,631) = 0.484 | *p* = .709 | n.a. |
| **RSQ-D - *Distraction*** | | |  |
| Time | F(1,631) = 64.456 | *p* < .001 |  |
| Group | F(3,631) = 2.052 | *p* = .106 |  |
| Time x Group | F(3,631) = 5.101 | *p* = .002 | guided iSMT ↑, unguided iSMT ↑ |

Note: pooled test statistics are derived from applying Rubin’s rules to the ten imputed data sets; significant interaction effects = changes of scores within each study arm across time (from t1 to t2); ↓ indicates a significant decrease in the score over time; ↑ indicates a significant increase in the score over time; DASS-21 = Depression-Anxiety-Stress Scale; ERQ = Emotion Regulation Questionnaire; RSQ-D = Responses Styles Questionnaire; n.a. = not applicable.
